# Flaviviruses as a Cause of Undifferentiated Fever in Sindh Province, Pakistan: A Preliminary Report

**DOI:** 10.3389/fpubh.2016.00008

**Published:** 2016-02-16

**Authors:** Erum Khan, Joveria Q. Farooqi, Kelli L. Barr, Dhani Prakoso, Amna Nasir, Akbar Kanji, Sadia Shakoor, Faisal Riaz Malik, Rumina Hasan, John A. Lednicky, Maureen T. Long

**Affiliations:** ^1^Department of Pathology, Aga Khan University, Karachi, Pakistan; ^2^Department of Microbiology, Aga Khan University, Karachi, Pakistan; ^3^Emerging Diseases and Arbopathogens Research and Testing Laboratory, Department of Infectious Diseases and Pathology, University of Florida, Gainesville, FL, USA; ^4^Department of Environmental and Global Health, Emerging Pathogens Institute, University of Florida, Gainesville, FL, USA

**Keywords:** arbovirus, flavivirus, dengue viruses, Japanese encephalitis virus, West Nile virus, transboundary diseases

## Abstract

Arboviral diseases are expanding worldwide, yet global surveillance is often limited due to diplomatic and cultural barriers between nations. With human encroachment into new habitats, mosquito-borne viruses are also invading new areas. The actual prevalence of expanding arboviruses is unknown in Pakistan due to inappropriate diagnosis and poor testing for arboviral diseases. The primary objective of this study was to document evidence of flavivirus infections as the cause of undifferentiated fever in Pakistan. Through a cooperative effort between the USA and Pakistan, patient exposure to dengue virus (DENV), West Nile virus (WNV), and Japanese encephalitis virus (JEV) was examined in Sindh Province for the first time in decades. Initial results from the 2015 arbovirus season consisting of a cross-sectional study of 467 patients in 5 sites, DENV NS1 antigen was identified in 63 of the screened subjects, WNV IgM antibodies in 16 patients, and JEV IgM antibodies in 32 patients. In addition, a number of practical findings were made including (1) *in silico* optimization of RT-PCR primers for flavivirus strains circulating in the Middle East, (2) shipping and storage of RT-PCR master mix and other reagents at ambient temperature, (3) Smart phone applications for the collection of data in areas with limited infrastructure, and (4) fast and reliable shipping for transport of reagents and specimens to and from the Middle East. Furthermore, this work is producing a group of highly trained local scientists and medical professionals disseminating modern scientific methods and more accurate diagnostic procedures to the community.

## Introduction

Political boundaries do not constrain human, animal, or plant diseases. As humans and animals migrate across these borders, their associated diseases relocate in tandem. Newly encroaching diseases are often overlooked because of limited surveillance, political motivation, resources, and infrastructure (1). Collaboration of health and scientific professionals from different countries occurs in a politically neutral atmosphere that can have rapid positive and sustainable impacts on human and animal health and the control of emerging diseases (2, 3). The growth in scientific personnel and infrastructure is essential to decrease the movement of diseases that threaten public health (1, 4–7). Many outbreaks since 1990 like prion disease in the UK, West Nile virus (WNV) in the America’s, and avian H5N2 in Canada and the US have been economically destabilizing and highlight the need for transboundary collaboration (8).

In the past decade, mosquito-borne viral diseases have emerged in many new locales, rapidly attaining endemic status. In Pakistan, arboviral diseases are frequently overlooked or misdiagnosed because of the vague symptoms and extensive differential diagnoses, which overlap with many other pathologies, such as Crimean-Congo Hemorrhagic fever (CCHF), malaria, hepatitis C virus infection, Alkhurma virus, Kyasanur forest virus disease, rickettsiosis, ehrlichiosis, leptospirosis, typhoid fever, meningococcemia, borreliosis, Q fever, and influenza. Furthermore, manifestations of arboviral disease mimic other febrile diseases and severe disease can present as a hemorrhagic illness [dengue virus (DENV), yellow fever virus, Zika virus, and Lassa fever virus], neurological disease (WNV, DENV), or arthritis (chikungunya virus, Zika virus, and DENV) (9, 10). Because vaccines or antivirals do not exist for most of these viruses, surveillance becomes an essential part of control *via* detection and communication. The cornerstone of active and passive surveillance is accurate diagnostic assessment (9, 10).

There has been limited published data for arbovirus surveillance in Pakistan. Historically, only the presence of DENV subtypes 1 and 2 were detected in isolated outbreaks in Pakistan in the twentieth century (11, 12). Since 2005, all four subtypes of DENV have spread throughout the country (12–15). In neighboring Punjab province, the seroprevalence of DENV in patients was 42.63% in 2013 (16). The WHO lists Japanese encephalitis virus (JEV) as active in Pakistan (17), although most reports still indicate JEV activity mostly along the northern Pakistan–India border (10, 18–20). JEV is likely circulating in Pakistan at this time, but limited information exists regarding the actual disease burden JEV contributes to human health in Pakistan. In the early 2000s, 25% of the Pakistani military personnel who tested seropositive for JEV demonstrated cross-reactivity with WNV and thus a true determination of infection could not be verified (21). WNV has been detected in Pakistan since 1980s. Epidemiological work performed 20 years ago indicated that WNV antibodies were present in over 40% of the human population in Punjab province (21). Recently, a 55% seropositivity rate was detected in horses in Punjab province (18, 20–24). This high seroprevalence in horses suggests that WNV is also circulating in humans.

Described here are the initial data of a biological engagement program (BEP) implemented between Pakistan (Aga Khan University, Karachi, Pakistan) and the US (University of Florida, Gainesville, FL, USA) to perform a multisite study examining possible arboviral causes of febrile disease in citizens of Sindh province. *Via* a “train the trainer” format, this project aimed to provide Pakistani collaborators with training for virus surveillance and diagnostics in order to assess the prevalence of flaviviruses (DENV, WNV, and JEV) in Pakistan. The primary objective of this study was to document evidence of the above mentioned viral infections as causes of undifferentiated fever in order to build capacity for laboratory diagnosis and surveillance within Pakistan.

## Materials and Methods

**Figure 1:**
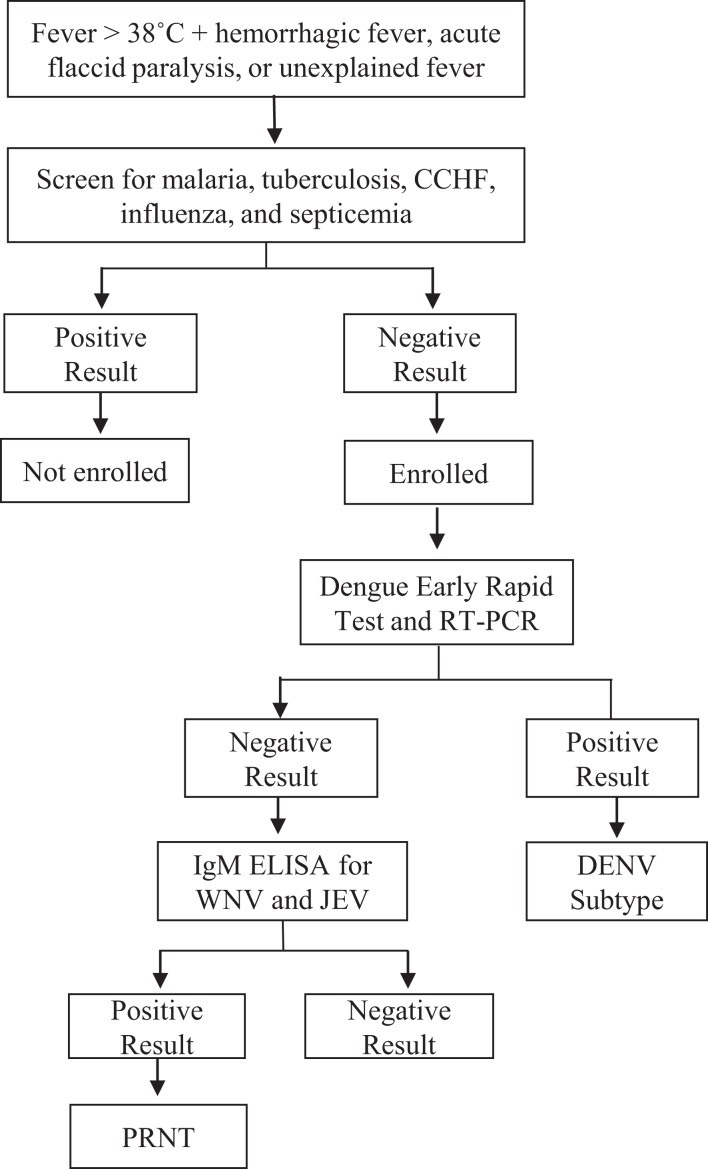
**Work flow for flavivirus exposure screening of enrolled patient samples**.

A cross-sectional, observational study was performed to identify which arboviruses (DENV, WNV, and JEV) were the cause of acute undifferentiated febrile illness in selected basic health units and/or district hospitals of the Sindh region of Pakistan. A total of 1,000 patients (250/year) patients were targeted for enrollment under informed consent procedures that were reviewed and approved by the Ethics Review Committee, Aga Khan University (#3183-PAT-ERC-14) and the Institutional Review Board, University of Florida (#201500908). All enrolled subjects gave written informed consent in accordance with the Declaration of Helsinki. Patients were recruited with a case definition developed by the WHO and modified by the Pakistan Ministry of Health to incorporate syndromic findings of acute hemorrhagic fever, acute flaccid paralysis, and unexplained fever (25). Patient enrollment was performed during the monsoon season (May–October) during 2015. All patients, males and females between 10 and 50 years age meeting the case definition on the day of enrollment, were eligible for the study. Patients younger than 10 and older than 50 years of age and patients who tested positive for CCHV, influenza, malaria, tuberculosis, and bacterial septicemia during routine hospital admittance procedures were excluded (Figure 1). Briefly, all patients were tested for DENV antigen unless affected primarily by neurological abnormalities. If positive, serum was tested for DENV subtype by RT-PCR. All negative sera were tested *via* IgM capture ELISA for JEV and WNV.

### Study Sites

**Figure 2:**
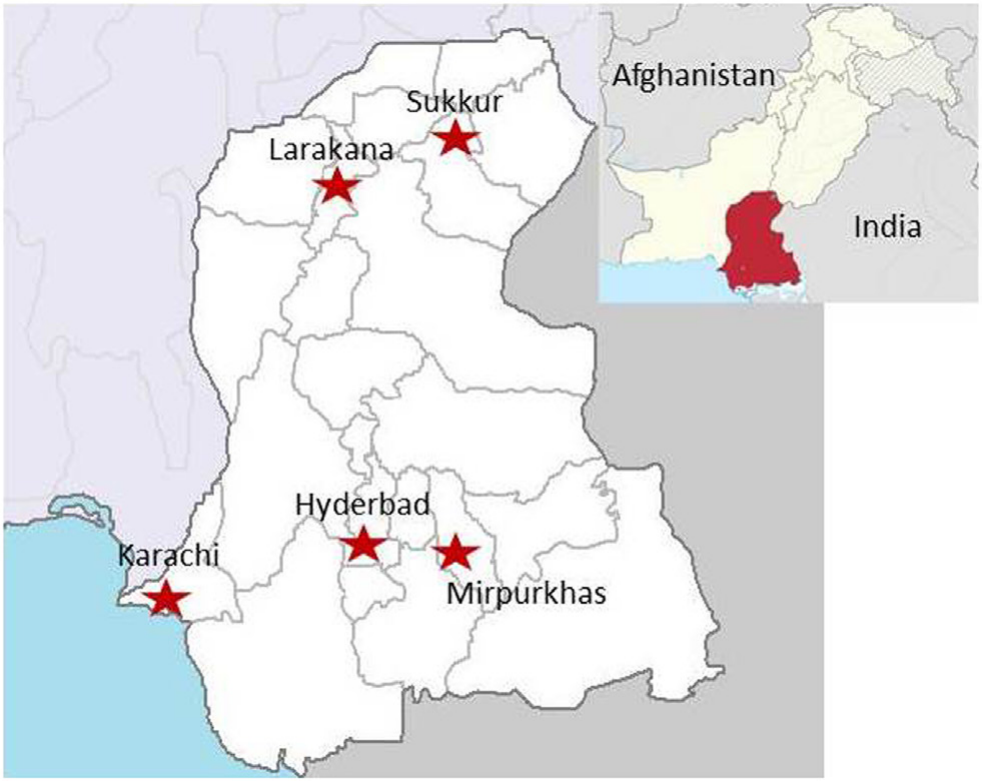
**Locations of study sites throughout Sindh, Pakistan**. (Image obtained at https://en.wikipedia.org/wiki/Sindh#/media/File:Sindh_in_Pakistan_%28claims_hatched%29.svg)

Five study sites were established and personnel trained throughout the Sindh province in Pakistan (Figure 2). These sites included four medical colleges including Ghulam Mohammad Mahar Medical College (Sukkur, Pakistan), CMC Teaching Hospital (Larkana, Pakistan), and Muhammed Medical College Hospital (Mirpurkhas, Pakistan). Enrollment of study subjects was also established at a civil hospital in Hyderabad, Pakistan.

### Data Collection and Processing Procedures

Originally, for communication within Pakistan between sites, networked computers were planned as the primary mode of reporting of test results. Connectivity was found to be a major issue; even if access was available, there were frequent interruptions and limited technological support. Android mobile phones provided an alternative for surveillance data collection and transmission (Epicollect^®^, http://www.epicollect.net/, Wellcome Trust, Imperial College London). At the study sites, patient information was collected on hard copy forms and de-identified data were stored on smart phones by a dedicated to a study medical officer and synced daily to a secure homepage on the *Epicollect* website.

### Antigen and Antibody Screening Tests

Standard operating procedures were developed that follow published WHO/CDC guidelines or publications that were optimized for detecting Asian variants of arbovirus (25). Primary DENV screening in patients was performed using a commercially available antigen capture test (Panbio Dengue Early Rapid Test, Alere, Waltham, MA, USA). IgM capture ELISA testing was performed using commercial assays for WNV and JEV (InBios, Seattle, WA, USA) following the manufacturer’s instructions.

### Real-Time PCR

For detection of nucleic acids of DENV, primer sequences were constructed for stains circulating in Pakistan *via* addition of degenerate nucleotides (Table 1) (26). Primers, standards, and controls were developed using synthetic DNA targets of the various portions of the viral genomes (Table 2) and real-time PCR was performed using a commercial master mix (BioRad iTaq Universal Probes Supermix, BioRad, Hercules, CA, USA) and a commercial real-time PCR machine (BioRad CFX 96, BioRad). All enrolled patients were screened for DENV *via* RT-PCR to identify DENV subtype.

**Table 1:** **RT-PCR primers and probes used for the detection of Dengue virus**.

	Forward 5′–3′	Reverse 5′–3′	Probe 5′–3′
DENV 1	CAAAAGGAAGTCGYGCWATA	CTGAGTGAATTCTCTCTRCTRAAC	FAM–CATGTGGYTGGGAGCRCGC–BHQ-1
DENV 2	CAGGYTATGGCACYRTCACRAT	CCATYTGCAGCARCACSATCTC	FAM–CTCYCCRAGAACGGGCCTMGACTTCAA–BHQ-1
DENV 3	GGACTRGACACACGCACCCA	CATGTCTCTACCTTCTCGACTTGYCT	FAM–ACCTGGATGTCGGCTGAAGGAGCTTG–BHQ-1
DENV 4	TYRTYCTAATGATGCTRGTCG	TCCACCYGAGACTCCTTCCA	FAM–CGGAATGCGATGCGTAGGRGTAGGRA–BHQ-1

**Table 2:** **Synthetic DNA targets used for RT-PCR**.

	Target gene	Target sequence
DENV 1	NS5	TTCGGAAAGGCAAAAGGAAGTCGTGCTATATGGTACATGTGGCTGGGAGCACGCTTTCTAGAGTTCGAAGCTCTTGGTTTCATGAACGAAGATCACTGGTTCAGCAGAGAGAATTCACTCAGCGGAGTGGAA
DENV 2	E	GCAGAGTTGACAGGCTATGGCACTGTCACGATGGAGTGCTCTCCGAGAACGGGCCTCGACTTCAATGAGATGGTGTTGCTGCAAATGGAAAATAAAGC
DENV 3	prM	TGTCGGCATGGGACTGGACACACGCACCCAAACCTGGATGTCGGCTGAAGGAGCTTGGAGGCAAGTCGAGAAGGTAGAGACATGGGCCCTTAGG
DENV 4	prM	ACTGTTTTCTTTGTCCTAATGATGCTAGTCGCCCCATCCTACGGAATGCGATGCGTAGGGGTAGGGAACAGAGACTTTGTGGAAGGAGTCTCGGGTGGAGCATGGGTCG

## Results

### Antigen and Antibody Screening Tests

As of this time, the five sites have enrolled a total of 467 participants. DENV antigen, using the DENV NS1 Early Rapid Test, was found in 63 of the enrolled patients (Table 3). WNV IgM antibodies were detected in 16 of 241 screened individuals and JEV IgM antibodies in 32 of 414 screened patients (Table 3). DENV was detected at all sights except Sukkur. Karachi had the highest DENV exposure with DENV antigen detected in 53 of 176 screened patients. Febrile patients with WNV exposure were found at four sites with eight of the patients living in Karachi, two in Hyderabad, two in Larkana, and one in Mirpurkhas (Table 4). JEV exposure was identified in 21 patients living in Karachi, 6 in Hyderabad, 1 in Mirpurkhas, 1 in Sukkur, and 1 patient in Larkana (Table 4).

**Table 3:** **Patients testing positive for exposure to dengue virus (DENV), West Nile virus (WNV), Japanese encephalitis virus (JEV), or flavivirus (JEV–WNV cross-reactive) detected in patients enrolled in five study sites in Sind Province, Pakistan**.

	Positive	Negative	Inconclusive/borderline	Total samples	% Positive
JEV	32	367	16	414	7.73
WNV	16	211	14	241	6.64
DENV	63	404	0	467	13.49
Flavivirus	32	382	0	414	7.73

**Table 4:** **Patients testing positive for exposure to dengue virus (DENV), West Nile virus (WNV), and Japanese encephalitis virus (JEV) at each study site**.

Study site	DENV	WNV	JEV
Karachi	53	8	21
Hyderabad	3	2	6
Mirpurkhas	6	4	1
Sukkur	0	0	1
Larkana	1	2	3

A number of inconclusive samples were obtained from study subjects (Table 3). A high signal:noise ratio and cross-reactivity were factors that prevented adequate interpretation. In 16 of the 414 patients screened for JEV, the ELISA results fell above background noise and just below IgM positive. The WNV assays resulted in 14 of 241 samples with similar inconclusive readings. Cross-reactivity between the WNV and JEV ELISA assays was also an issue with 32 (up to 13%) samples that tested positive for both WNV and JEV exposure.

### Real-Time PCR

**Figure 3:**
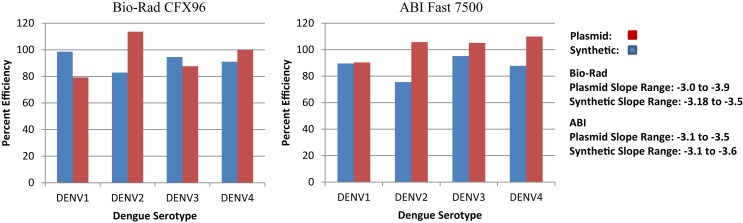
**RT-PCR efficiencies of synthetic DNA targets of dengue virus compared to the RT-PCR efficiencies of conventional dengue virus DNA from plasmids**.

Synthetic targets were developed for use as standards and controls for the RT-PCR platform (Table 2). Targets were optimized to perform as well as or better than conventional plasmids (Figure 3) and the difference in percent efficiency was 10% or less for DENV1 and 3 and <20% for DENV2 and DEN4. In addition, we found that our plasmid controls were frequently >100% in efficiency (slope <−3.5).

## Discussion

Arboviral infections have a global distribution; however, the burden of viral agents varies in different geographical regions. The true burden and epidemiology of arboviruses in Pakistan are not known as many of these infections, which present initially as a vague febrile illness, are often misdiagnosed.

The expansion of DENV in Pakistan has been notable in its intensity. Recently, DENV emerged in Karachi in Sindh, Pakistan affecting 3,640 patients with an estimated 40 deaths (12–14). WNV is an arbovirus undergoing expansion throughout the world. While it is similar to JEV in terms of syndromes, most human JEV exposure and illness is described in children (27). For WNV, people over the age of 55 years have the highest risk factors for neurological syndromes during the virus’ recent expansion throughout Europe (28). Comparative analysis of risk for concomitantly circulating JEV and WNV has not been performed. Recent evidence demonstrates a high amount of WNV activity in Pakistan in horses (20); however, there is limited information regarding recent human exposure and, to the best of our knowledge, the most commonly reported cause of neurological arboviral disease would likely be JEV (20).

Dengue virus was the most frequently identified and widespread flavivirus detected in enrolled patients. These data show that DENV was detected in nearly one-third of all patients in Karachi while it was found at much lower rates in other locations. This is most likely due to the fact that Karachi is an expansive urban environment with the ideal climate for the DENV vector *Aedes aegypti*. The other four sites displayed a much lower human exposure of arboviral diseases than Karachi, most likely because suitable conditions for the vector are absent. Flavivirus exposure was detected in only one patient living in Sukkur. This may be a function of vector biology or the low number of screened patients. Sukkur is a small, sparsely populated district where the climate is very hot, windy, and dry, which is not suited to most mosquitoes.

Commercial assays make field work in limited areas feasible. They are easy to use and results are easy to interpret. However, especially for WNV, the assay is expensive. As expected, there was significant cross-reactivity between WNV and JEV when using IgM ELISA kits. If JEV and WNV ELISA data are grouped as “flavivirus exposed,” roughly 13% of screened patients were positive. Consistent with the algorithm, all of the WNV and JEV samples will be tested by plaque reduction neutralization test (PRNT) to confirm which disease agent was present.

This cooperative BEP with Pakistan shows that scientific and medical projects can be successful and rapidly established between academic professional of countries that have limited political relations. Relationships were enhanced through early discussions between partners that included cultural and scientific barriers to health to optimize scientific training and develop methods for clinical surveillance. Barriers to recruitment of patients included lack of understanding of basic clinical signs of arboviral diseases on the part of health-care professionals. This was remedied *via* focused training study personnel who were employed locally.

Supply and communication lines also needed to be addressed by investment of research time into questions that challenge issues of data transfer and cold-chain supply without the need for development of entirely new technologies. For assay development, reagents and assays were developed to obviate the need for a cold-chain. In particular, it was determined that the BioRad chemistries could be shipped at ambient temperature and was confirmed by sending the reagents from the UF laboratory to the Aga Khan laboratory. When using plasmid technology, problems arose with reagent stability if shipping was interrupted (>3 weeks); thus, commercially manufactured real-time targets were used. These targets proved to be highly stable and exceptionally cost-effective. This also decreased the need to send supplies on dry ice that offered a substantial decrease in shipping costs and increased the availability of other forms of freight.

One of the most common challenges faced in the establishment of this project centered on limited communication to establish the needs of the US–Pakistan collaborators themselves. Most communications were written and did not include face-to-face fact finding before embarking on training and teaching sessions. In addition, problems with computer-based communication and travel (both local and abroad) delayed development of laboratory expertise. At the study sites, basic mobile phone technology was relied upon to share patient cases with infectious disease experts and the availability of a freely hosted website greatly facilitated this. Differences in compliance requirements at both University and government levels posed significant obstacles for transfer of medical technology.

Despite these issues, many goals have been attained within the first year of establishment of this project; a collaborative environment between a US-based University and a Pakistan-based University for the purposes of research and training exchange and a multisite network for arbovirus surveillance in humans across one of the largest and most populous provinces of Pakistan were established. US partners gained an understanding of the climatic, geographic, and cultural landscape of Pakistan and how this may contribute to arbovirus expansion. Finally, for the first time, preliminary assessment of the variety of several important arboviruses was determined indicating the need for continued surveillance and testing.

## Author Contributions

The following authors contributed to this manuscript in the following ways: contribution to the conception and design of the work: ML, EK, and KB; acquisition, analysis, and interpretation of data: ML, EK, DP, KB, AK, AN, JF, SS, FM, RH, and JL; drafting, editing, revising, and approving drafts: ML, KB, EK, DP, AK, AN, JF, SS, FM, RH, JL. ML, EK, and JL. All agreed to be accountable for all aspects of the work.

## Conflict of Interest Statement

The authors declare that the research was conducted in the absence of any commercial or financial relationships that could be construed as a potential conflict of interest.
